# Efficacy and safety of acupoint catgut-embedding for ulcerative colitis: A protocol for systematic review and meta-analysis

**DOI:** 10.1097/MD.0000000000022658

**Published:** 2020-10-16

**Authors:** Mingsheng Chen, Ping Xin, Kaidi Feng, Tianyu Zhao, Xiangdong Yang

**Affiliations:** a Clinical School of Chengdu University of Traditional Chinese Medicine, Chengdu, Sichuan province.; b Tianjin University of Traditional Chinese Medicine, Tianjin; c Chengdu Anorectal Hospital, Chengdu, Sichuan Province, China.

**Keywords:** acupoint catgut-embedding, protocol, systematic review, ulcerative colitis

## Abstract

**Background:**

Ulcerative colitis (UC) is a refractory intestinal disease prone to recurrent attacks, with a high rate of canceration, which seriously affects life treatment. Routine treatment has disadvantages such as long course of treatment, high cost, easy recurrence and limited effectiveness. Clinical practice shows that acupoint catgut embedding therapy has certain therapeutic advantages but lacks evidence of evidence-based medicine. The purpose of this study is to systematically study the effectiveness and safety of acupoint catgut embedding for ulcerative colitis.

**Methods:**

Retrieve English database (PubMed, Embase, Web of Science, the Cochrane Library) and Chinese database (CNKI, CDDB, CQVIP, CBM) by computer, and manually retrieve Baidu and Google Academy for randomized controlled trials (RCTs) of acupoint catgut embedding therapy for ulcerative colitis from the time of construction of database to September 2020. Two researchers independently extracted data and evaluated the quality of the literature included in the study, and used RevMan 5.3 software for meta-analysis of the included literature.

**Result:**

The study evaluated the effectiveness and safety of acupoint catgut embedding for ulcerative colitis through efficiency, symptom score, colonoscopy score, mucosal healing rate, recurrence rate, incidence of adverse reactions, etc.

**Conclusion:**

This study will provide reliable evidence-based evidence for clinical application of acupoint catgut embedding therapy for ulcerative colitis.

**OSF Registration number:**

DOI 10.17605/ OSF.IO / 7T4QV.

## 1 Introduction

Ulcerative colitis (UC) refers to a chronic inflammatory disease involving the rectum and colonic mucosa, which is a lifelong disease caused by the interaction of genetic and environmental factors.^[1]^ In the past 20 years, the incidence of UC has continued to rise, with great differences between eastern and Western countries, ranging from 0.97 to 57.9 per 100,000 in Europe, 8.8 to 23.14 per 100,000 in North America, and 0.15 to 6.5 per 100,000 in Asia and the Middle East.^[2]^ The clinical manifestations are abdominal pain, diarrhea, mucopurulent, and bloody stools, etc., which are characterized by recurrence and remission.^[3]^ Colorectal cancer is an important complication of long-term ulcerative colitis, with a high mortality.^[4]^ At present, conventional treatment drugs include aminosalicylic acid, adrenocortical hormones, immunosuppressive agents, new immunomodulators, anticoagulants, microbial agents, etc.^[5]^ However, long-term use of these drugs reduces their efficacy and is usually accompanied by variable side effects, such as diarrhea, nausea, vomiting, headache, and osteoporosis,^[6]^ so new treatment options are urgently needed. At present, more and more TCM treatment schemes are applied to the treatment of ulcerative colitis.^[7]^

Acupoint catgut embedding therapy is a traditional external treatment method in China, which has been widely used in the treatment of hypertension, diabetes, anal fissure, acute and chronic pain, and other diseases,^[8–11]^ with significant efficacy, low cost, low adverse reactions, and other advantages. Clinical studies have confirmed that the immune level of patients with ulcerative colitis is lower than that of normal people,^[12]^ while clinical observation has found that acupoint catgut embedding therapy for ulcerative colitis has the effect of reducing inflammatory response, improving immunity, promoting mucosal regeneration,^[13]^ and has definite curative effect. At present, a number of randomized controlled studies have also confirmed this viewpoint,^[14–16]^ but there are differences in research programs and efficacy of each clinical trial, resulting in uneven research results, which to some extent affects the promotion of this treatment. Therefore, this research plan systematically evaluates the efficacy and safety of acupoint catgut embedding in the treatment of ulcerative colitis, and provides reliable reference basis for clinical application of acupoint catgut embedding in the treatment of ulcerative colitis.

## 2 Methods

### 2.1 Protocol register

This protocol of systematic review and meta-analysis has been drafted under the guidance of the preferred reporting items for systematic reviews and meta-analyses protocols (PRISMA-P). Moreover, it has been registered on open science framework (OSF) on September 8, 2020. (Registration number: DOI 10.17605 / OSF.IO / 7T4QV).

### 2.2 Ethics

Since this is a protocol with no patient recruitment and personal information collection, the approval of the ethics committee is not required.

### 2.3 Eligibility criteria

#### 2.3.1 Types of studies

We will collect all available randomized controlled trials (RCTs) on acupoint catgut embedding for ulcerative colitis, regardless of blinding, publication status, region, but Language will be restricted to China and English.

#### 2.3.2 Study subjects

Patients with definite diagnosis of ulcerative colitis (refer to the diagnostic criteria of ulcerative colitis in the Consensus Opinions on Diagnosis and Treatment Standards of Inflammatory Bowel Disease in China published by the Chinese Society of Traditional Chinese Medicine in 2007^[17]^), regardless of nationality, race, age, sex, course of disease, etc.

#### 2.3.3 Interventions

The treatment group was treated with acupoint catgut embedding alone, excluding the study combined with other therapies, while the control group was treated with conventional drugs. The treatment group did not restrict the acupoint selection scheme, frequency of treatment and course of treatment, while the control group did not restrict the type of drugs, dosage form, frequency, and course of treatment.

#### 2.3.4 Outcome indicators

(1) Primary outcome:

1. the overall effective rate, referring to the diagnostic criteria of ulcerative colitis in the Consensus Opinions on Diagnosis and Treatment Standards of Inflammatory Bowel Disease in China published by the Chinese Society of Traditional Chinese Medicine in 2007,^[17]^ the total effective rate = (number of complete remission + effective number)/total number ∗100%. Among them, complete remission represents the disappearance of clinical symptoms, and the mucosa is found to be roughly normal by colonoscopy reexamination;
2. effective represents the basic disappearance of clinical symptoms, mild inflammation of mucosa or pseudopolyp formation by colonoscopy reexamination;
3. ineffective represents no improvement of clinical symptoms, endoscopy, and pathological examination results after treatment.

(2) Secondary outcomes:

1. symptom score^[18]^;
2. colonoscopic score (refer to Baron endoscopy score^[19]^);
3. mucosal healing rate (refer to Mayo index endoscopy score^[20]^);
4. serum bloody test (such as Antineutrophil cytoplasmic antibodies, ANCA);
5. recurrence rate;
6. incidence of adverse reactions.

### 2.4 Exclusion criteria

1. Repeated published papers;
2. Papers whose publications are abstracts or whose data is incomplete and the complete data cannot be obtained after contacting the authors;
3. Studies with obvious data errors;
4. Literatures assessed as high risk of bias by randomization or allocation of concealment^[21]^;
5. Studies in which treatment groups with other TCM therapies, such as acupuncture, moxibustion, traditional Chinese medicine compound, etc.
6. Literatures without relevant outcome indicators.

### 2.5 Retrieval strategy

Chinese databases were retrieved using “acupoint catgut embedding”(xue wei mai xian) and “ulcerative colitis”(kui yang xing jie chang yan) as Chinese search terms, including China National Knowledge Infrastructure (CNKI), Wanfang Data Knowledge Service Platform, VIP Database for Chinese Technical Periodicals (VIP), and China Biomedical Database. The English databases, including PubMed, EMBASE, Web of Science, the Cochrane Library, were retrieved with the English search terms of “acupoint catgut-embedding therapy”, “Acupuncture point catgut embedding”, “Ulcerative colitis”, “Colitis Gravis”. In addition, manual searches were conducted in Baidu and Google Academy. The retrieval time was from the establishment of the database to September 2020, during which all the domestic and foreign literatures on the treatment of ulcerative colitis by acupoint catgut embedding were collected. Taking PubMed as an example, the retrieval strategy is shown in Table 1.

**Table 1:**
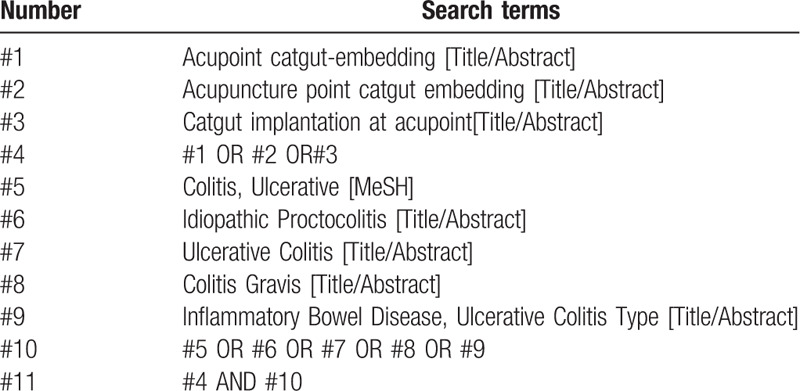
Search strategy in PubMed database.

### 2.6 Data screening and extraction

Referring to the method of research selection in version 5.0 of the Cochrane collaboration Network system Evaluator Manual, according to the PRISMA flow chart, the 2 researchers used the EndNote X9 document management software to independently screen and check the literature according to the above inclusion and exclusion criteria, and check each other, if there were different opinions, negotiate with a third party to resolve the differences. At the same time, Excel 2013 was used to extract relevant information, including:

1. Clinical studies (title, first author, publication year, sample size, sex ratio, average age, average course of disease);
2. Interventions (acupoint selection scheme, frequency, course of treatment in treatment group; drug type, dosage form, frequency, course of treatment in treatment group);
3. Risk bias assessment elements in randomized controlled trials;
4. Outcome indicators.

**Figure 1:**
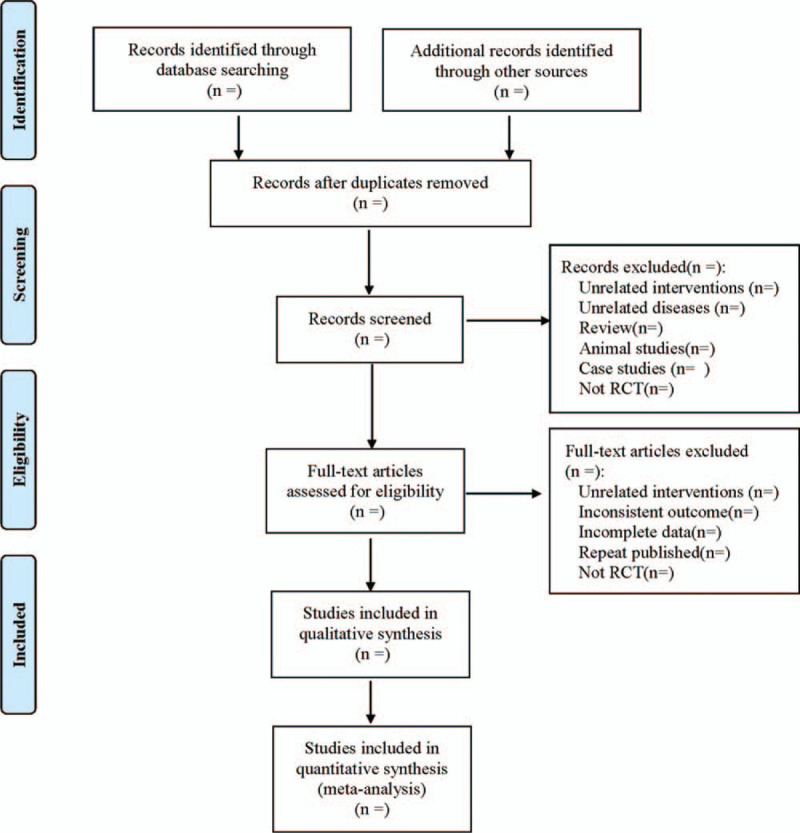
The process of literature filtering.

The literature screening process is shown in Figure 1.

### 2.7 Literature quality assessment

Use the Cochrane collaborations tool for assessing risk of bias to do the risk of bias assessment of included studies. According to the performance of the included literatures in the above evaluation items, 2 researchers will give judgments like low risk, unclear or high risk judgments one by one, and cross-check after completion respectively. In case of any disagreement, discussion will be carried out. If no agreement can be reached between the 2, discussion will be made with the researchers in the third party.

### 2.8 Statistical analysis

#### 2.8.1 Data analysis and processing

The RevMan 5.3 software provided by the Cochrane Collaboration will be used for statistical analysis.

1. Relative risk (RR) is selected as the statistic for the dichotomous variable. For continuous variables, weighted mean difference (WMD) is selected when the tools and units of measurement indicators are the same, standardized mean difference (SMD) is selected with different tools or units of measurement, and all the above are represented by effect value and 95% confidence interval (CI).
2. Heterogeneity test: Q test is used to qualitatively determine inter-study heterogeneity. If *P* ≥ .1, there is no inter-study heterogeneity; If *P* < .1, it indicate inter-study heterogeneity. At the same time, *I*^*2*^ value is used to quantitatively evaluate the inter-study heterogeneity. If *I*^*2*^ ≤ 50%, the heterogeneity is considered to be good, and the fixed-effect model is adopted. If *I*^*2*^ > 50%, it is considered to be significant heterogeneity, the source of heterogeneity will be explored through subgroup analysis or sensitivity analysis. If there is no obvious clinical or methodological heterogeneity, it will be considered as statistical heterogeneity, and the random-effect model will be used for analysis. Descriptive analysis will be used if there is significant clinical heterogeneity between the 2 groups and subgroup analysis is not available.

#### 2.8.2 Dealing with missing data

If there is missing data in the article, contact the author via email for additional information. If the author cannot be contacted, or the author has lost relevant data, descriptive analysis will be conducted instead of meta-analysis.

#### 2.8.3 Subgroup analysis

Subgroup analysis can be carried out according to the condition, which can be divided into active period and remission period; subgroup analysis can be carried out according to the course of treatment; subgroup analysis can be carried out according to the different drug treatment regimens in the control group.

#### 2.8.4 Sensitivity analysis

In order to test the stability of meta-analysis results of indicators, a one-by-one elimination method will be adopted for sensitivity analysis.

#### 2.8.5 Assessment of reporting biases

Funnel plots were used to assess publication bias if no fewer than 10 studies were included in an outcome measure. Moreover, Eggers and Beggs test were used for the evaluation of potential publication bias.

#### 2.8.6 Evidence quality evaluation

The Grading of Recommendations Assessment, Development, and Evaluation (GRADE) will be used to assess the quality of evidence. It contains 5 domains (bias risk, consistency, directness, precision, and publication bias). And the quality of evidence will be rated as high, moderate, low, and very low.

## 3 Discussion

Ulcerative colitis belongs to the category of “Chang pi”(hematochezia), “Li ji”(dysentery), “Chang feng”(hematochezia), “Zang du”(anal cryptitis), and “Xia li”(diarrhea) in TCM. Its basic pathogenesis is heat and humidity accumulating in the intestines and qi stagnation and obstruction of collaterals.^[22]^ The pathogenesis of the disease is still unclear in modern medicine, which may be a complex result^[23]^ of intestinal mucosal immune dysfunction caused by genetic, environmental, infectious, psychological, intestinal mucosal barrier function defects and other factors. Although there are many treatment options at present, the curative effect is limited and the condition is repeated. The patients should not only endure the pain of the disease itself, but also suffer from various adverse reactions of treatment, which seriously disturb the physical and mental health of the patients.

Acupoint catgut embedding therapy is the extension and development of acupuncture and moxibustion therapy. Under the guidance of meridian theory, catgut or absorbable surgical thread is implanted into corresponding acupoints to produce a lasting, slow and stable acupuncture effect during the process of decomposition and absorption. Commonly used acupoints include ST36(Zusanli), ST25(Tianshu), BL25(Dachangshu), RN12(Zhongwan), BL20(Pishu), ST37(Shangjuxu), etc. ST36(Zusanli) has the effect of reinforcing the vital essence and strengthening the primordial qi, as well as enhancing immunity; ST25(Tianshu) has the effect of regulating qi and strengthening spleen, and relieving diarrhea with astringents; RN12(Zhongwan) has the effect of strengthening spleen and regulating stomach, and regulating qi; BL20(Pishu) has the effect of strengthening spleen, removing dampness and anti-diarrhea; BL25(Dachangshu) and ST37(Shangjuxu) have the effect of promoting qi and activating blood circulation, and keeping balance of yin and yang. Clinically, acupoint selection according to syndrome differentiation can achieve the functions of dredging meridians and collaterals, regulating qi and blood, and adjusting viscera. Modern studies have confirmed that ST36(Zusanli) and ST37(Shangjuxu) have the functions of regulating immunity and gastrointestinal smooth muscle movement, improving intestinal blood flow, promoting the neogenesis of diseased tissues and repairing ulcers,^[24]^ ST37(Shangjuxu), ST25(Tianshu), and BL25(Dachangshu) can regulate the expression of IL-17, **β**2AR and NF-_K_Bp65, and play an anti-inflammatory and mucosal repair role.^[25]^

Clinical studies have confirmed the efficacy of acupoint catgut embedding in the treatment of ulcerative colitis is reliable, however, this is not consistent with the evidence from RCTs. With the increasing number of clinical trials, it is urgent to systematically evaluate the efficacy of acupoint catgut embedding in the treatment of ulcerative colitis. In this study, we will summarize the latest evidence of the efficacy of acupoint catgut embedding in the treatment of ulcerative colitis. This work also provides useful evidence for determining whether acupoint catgut embedding therapy is effective and safe for patients with ulcerative colitis, which is beneficial to both clinical practice and health-related decision makers.

However, this systematic review has some limitations. There may be some clinical heterogeneity among the included studies in terms of acupoints, the material of embedding thread, and the degree of patients illness. In addition, the course of treatment varies among patients, which may have an impact on the outcome. Due to the limitation of language ability, we only search English and Chinese literature, and may ignore studies or reports in other languages.

## Author contributions

**Data curation:** Ming Sheng Chen, Ping Xin.

**Funding acquisition:** Xiangdong Yang.

**Investigation:** Xiangdong Yang.

**Resources:** Kaidi Feng.

**Software:** Tianyu Zhao.

**Writing – original draft:** Ming Sheng Chen, Ping Xin.

**Writing – review & editing:** Xiangdong Yang.
